# Potentials of invasive *Bidens pilosa*, *Conyza bonariensis* and *Parthenium hysterophorus* species based on germination patterns and growth traits

**DOI:** 10.1371/journal.pone.0309568

**Published:** 2024-09-05

**Authors:** Rahmah Al-Qthanin, Asmaa M. Radwan, AbdElRaheim M. Donia, Mohamed A. Balah

**Affiliations:** 1 Department of Biology, College of Science, King Khalid University, Abha, Saudi Arabia; 2 Prince Sultan Bin-Abdul-Aziz for Environmental Research and Natural Resources Sustainability Center, King Khalid University, Abha, Saudi Arabia; 3 Faculty of Science, Girls Branch, Al-Azhar University, Cairo, Egypt; 4 Desert Research Center, Cairo, Egypt; NSW DPI: New South Wales Department of Primary Industries, AUSTRALIA

## Abstract

Invasive alien species drive extensive ecological changes and cause unexpected risks worldwide. Perceptive germination requirements and the growth function of invasive species are crucial for understanding their invasion and subsequent dissemination in various environmental conditions. Therefore, the germination response of invasive *Conyza bonariensis*, *Parthenium hysterophorus*, and *Bidens pilosa* of Asteraceae family were examined under alternating temperature regimes and some environmental factors. The prevailing germination ability occurs highest at moderate-temperature regimes at 20/30°C attained by 94.83% (*C*. *bonariensis*) and at 20/25 SS by 96.28% (*P*. *hysterophorus*) and high-temperature regimes at 25/30°C reached 92.94% (*B*. *pilosa*) respectively. The half germination percentage (G_50_) was -0.406 MPa and 2878.35 ppm (*B*. *pilosa*), -0.579 MPa and 2490.9 ppm (*C*. *bonariensis*), and*—*0.32 MPa and 2490.8 ppm (*P*. *hysterophorus*) affected by osmotic pressure and salt stress (NaCl) respectively. The highest growth plasticity characteristics were identified in total dry mass attained at 0.968 (*C*. *bonariensis*), 0.985 (*B*. *pilosa*) and 0.957 (*P*. *hysterophorus*) respectively. The relative growth, net assimilation and plasticity index appeared higher in both *B*. *pilosa*, and C. *bonariensis* than *P*. *hysterophorus* in the invaded area. In conclusion, germination and growth traits are precisely functional factors that correlate to invasion success under stressed conditions, and zones, and also lead to successful control plans for invasive species and ecological protection.

## Introduction

Invasive species (IS) are introduced organisms unintentionally or consciously into a new habitat that is not part of their normal geographic range [1]. They are responsible for disrupting ecosystems by posing numerous problems for native species [2] and threatening human health, and food supply [3]. The estimated loss from IAS worldwide was about One trillion and forty-four dollars which amounts to 5% of the GDP [4]. The successful invasion may be facilitated by biological and functional features that increase niche differences or provide invaders with competitive advantages [5,6]. These attributes can take in the prediction of invasiveness [7]. Therefore, measuring is necessary to prevent invasions and negative effects in the future [8]. Knowledge about plant germination and biology aids in determining their potential and facilitates their successful management [9,10].

A crucial step in species extension into new settings is the germination stage and plant invasions [11,12]. The significant environmental element to regulatory germination of seeds is temperature [13], and the establishment of populations as well as affecting the successful invasion [14,15]. This warming by temperature affects plant invasions directly and drives the ejection of the enemy [16].

Seed traits play a significant role in species distribution and preference for microhabitats [17]. The success of spreading throughout a range of broad temperatures where germination is possible [18]. The Most wide germination range of plants occurs between 0°C and 35°C [19]. Regarding Invasive species, they mostly germinate faster than and with a higher germination rate [20,21]. Invasive plants typically exhibit greater germination cues and germinate earlier or quicker than in their native range than non-invasive [22,23]. Thus, the distributions of the invasive grass are predicted using the germination pattern temperature relationship and region [24,25].

The invasion successes is influenced by functional features of developmental, physiological, and phenotypic plasticity, which differ across invading species at different stages [26,27]. It also includes prolonged periods of flowering and fruiting [28], as well as a higher specific area of leaf and growth rate [29], seed production and vegetative growth [30,31]. Under nutrient-enriched circumstances, Exotic invasive than native species have strong growth and survival [32]. A classic growth analysis decomposes relative growth rate (the relative increase in weight per day) into two components: net assimilation rate and leaf area ratio [33]. Seedling relative growth rate analysis is a powerful tool for understanding life-history traits because it combines aspects of species anatomy, morphology, and physiology [34]. Variation in RGR has even been described as ‘among the most important spectra of plant adaptations [35]. While the net assimilation rate (NAR) was strongly and positively associated with area-based photosynthetic rate and leaf nitrogen concentration can, therefore, be good predictors of species growth [36]. The ability of invasive plants to achieve higher relative growth rates (RGR) than their native counterparts has been widely documented [37]. In disturbed ecosystems, RGR is the most significant predictor of invasiveness and relates to physiological measures of invasiveness [38]. Stages and habitats have an impact on *Solanum elaeagnifolium* relative growth, the ratio of S/R (shoots to roots ratio), LAR (ratio of leave areas), and LMF (fraction of leave mass) [39]. Mass of seeds, juvenile period, and seed harvests are three characteristics that may be used to explain why Pinus species are invasive [40]. *P*. *angulata* is known for its greater plasticity, which describes its remarkable capacity to adapt to changing environmental circumstances and to invade a new community [41].

Asteraceae, is responsible for many of the most problematic invasive weeds [42,43]. An annual plant native to South America, *Bidens pilosa* L. (Asteraceae) is extensively distributed across the world area including tropical and subtropical [44], *Conyza bonariensis* is native to South America [45], and it is an opportunistic invader of subhumid, subtropical pastures [46]. *Parthenium hysterophorus* (Asteraceae family) originated in the Americas and currently invades over 40 countries across five continents [47].

In Egypt, there is inadequate information about invasive weed species ecology, behavior, suitable action and priority species in the invaded areas. On the other side, germination and growth measuring can be considered very important factors to predict their impacts and invasion abilities outside their geographical zones in the future. Predicting the invasiveness of IAS is necessarily to consider the climatic parameters [48]. So we hypothesized that the germination abilities and growth characteristics of Asteraceae invasive species are affected greatly by environmental conditions. These traits can characterize their spreading patterns and help in the prediction process for their future invasion of new areas. In this work, a series of experiments tested the germination abilities of invasive *B*. *pilosa*, *C*. *bonariensis* and *P*. *hysterophorus*, using 36 combinations of alternating temperature regimes and some environmental stress. In addition to the phenological traits and growth in the invaded lands to facilitate their management into the invaded areas and prevent their future invasion.

## Materials and methods

### Plant material collections

All the steps of experimentation on *Bidens pilosa*, *Conyza bonariensis* and *Parthenium hysterophorus* weeds, including the collection of plant material, are in compliance with relevant Institutional, National, and International guidelines. The studies were conducted in accordance with local legislation and with permissions from Desert Research Center and complied with the IUCN Policy Statement.

### Germination experimental process under alternating temperatures

Three invasive alien weed species Asteraceae including *Bidens pilosa* seeds were collected from Al-Kanter, Qalyubia governorate in August 2021, *Conyza bonariensis* seeds were collected from Al-Nubariya, Al-Beheria in October 2021, *Parthenium hysterophorus* seeds were collected in July 2021from Qutor, Al-Gharbia governorate in Egypt. These plants were identified as Voucher specimens CAIH-1214-B, CAIH-1215-C, and CAIH-1216P, respectively in the herbarium by Dr. Emad Abdelkhader Desert Research Center, Cairo, Egypt. Seed samples were dried under laboratory conditions and preserved at 4°C until used The experimental protocol of seed germination for Asteracea plants was implemented according to **Young et al.,** [49,50]. The tested temperature relates to the mean field conditions that start from 5 to 40°C into combinations of 36 alternating temperature regimes under 12/12h (light/dark) with a gradient every 5°C. These regimes are divided into five consequences split grouped of temperature according to **Pitcairn et al.,** [51] low temperature (5/5°C, 5/10°C, 10/10°C), moderately high and low temperature (5/15°C, 5/20°C, 5/25°C, 10/15°C, 10/20°C, 10/25°C, 10/30°C, 15/15°C, 15/20°C, 15/25°C, 15/30°C, 15/35°C, 20/20°C, 20/25°C, 20/30°C, 20/35°C, 25/25°C, 25/30°C), high temperature (20/40°C, 25/35°C, 25/40°C, 30/30°C, 30/35°C, 30/40°C), large range high and low temperature (5/30°C, 5/35°C, 5/40°C, 10/35°C, 10/40°C, 15/40°C), and extremely high temperature (35/35°C, 35/40°C, 40/40°C) respectively. Twenty-five seeds were sterilized with 1% sodium hypochlorite and placed in culture 9-cm dishes that contained wetted filter paper and placed in the corresponding temperature incubator. Four petri dish replicates for each temperature regime were settled randomly in the incubators set alternatively from 5 to 40°C under 12/12h (light/dark). The germination tests were repeated twice at least to avoid any errors. During 30 days, the number of seeds germination was identified for all species. The maximum germination percentages (G_max_) and G_rate_ as well as T_50_ were obtained after fitting the regression model to have an R^2^ value ≥ 0.9.

### Germination under environmental stress of selected invasive weeds

To test the water potentials effect on the selected species germination, A range of osmotic potentials of 0.0, -0.10, -0.25, -0.50, -1.00, and -1.50MPa prepared using PEG (Polyethylene glycol) based on **Michel** [52] **Michel and Radcliffe** [53]. The salt stress consequence was tested on species germination, an experiment was established using 0.0, 500, 1000, 2000, 3000, 4000, and 5000 ppm concentrations of NaCl [54,55], whereas, the salinity of Egypt’s soil is reflected in this concentration of salt. The species seeds were soaked in pH solutions from 1.00 to 12.0 levels according to **Burke et al.,** [56]. Then in Petri dishes, twenty-five sterilized seeds were placed in two filter pepper substrates wet with ten milliliters of the tested solution. These dishes were incubated for four weeks at 20/25°C, 20/30°C and 25/30°C, (the maximum temperature regime), under twelve hours light and dark for *P*. *hysterophorus*, *B*. *pilosa*, and *C*. *bonariensis* respectively, under observation until 30 days to recording germination.

### Growth traits of the invasive weeds in invaded lands

To study the phenology of *B*. *pilosa*, *C*. *bonariensis*, and *P*. *hysterophorus* invasive weeds, ten invaded sites were collected fifty plants at each life stage in Qalyubia, Al-Beheria, Al-Gharbia governorates of Egypt. These samples were sectioned into roots, shoots, leaves, and weighted. Leaf area was detected before drying at 80°C. The data of lengths and the number of leaves were recorded. The collected soil from the invaded sites was analyzed [57]. RGR was calculated According to **Cornelissen et al.,** [58], and NAR is determined according to **Gregory** [59]. The Plant traits including R/S (The ratio of root/shoot), SLA (the specific area of leaves), SSL (the length of specific stem) and RMF (the fraction of root mass) were determined [60]. The growth of invasive weeds was measured from dry weight accumulation [61]. The index of plasticity (PI) of each invasive species was calculated according to **Valladares et al.,** [62].

## Data analysis and statistics

The data of the cumulative germination percentage were taken to **Sigma Plot**®12.5 software for analysis using the model: G (germination %) = (G_max)_ /(1 + e [(-x-T_50)_] G_rate)_. Whereas x, and T _(50)_ are the time, and the required time for 50% inhibition (half-time) [51]. The nonlinear regression models according to **Evans and other** [63] and **Lu et al.,** [64] using **Sigma Plot**®12.5 software processed from cumulative germination as regression analysis with a three-parameter sigmoid function. The obtained data of germination percentage and growth traits (weights, lengths and areas) for 4 replications were entered statistics (ANOVA) into IPM SPSS 21, while, the Duncan test was chosen for multiple comparisons and to compare the differences within each species (p *≤* 0.05).

## Results and discussion

### Germination traits of invasive *B*. *pilosa*, *C*. *bonariensis*, and P. *hysterophorus* under alternating temperature regimes

Table 1 demonstrated that the optimum germination profile for *B*. *pilosa* species occurred at 25/30°C (high-temperature regime) by 92.94% and 30/35°C by 91.94%, followed by 20/30°C (moderate temperature regime) by 88.41% and 15/25°C by 86.80% respectively. Almost its germination was nonexistent over 5/5°C, 5/10°C, 5/40°C, 35/35°C and 40/40°C. The lengthy T_50_ was found at 5/15°C estimated by 23.27 days, and substantially identified at 10/15°C by 22.98 as well as the maximum G_rate_ was significantly detected at 25/30°C by 0.619. The optimum germination of *C*. *bonariensis* (G _max_) was found when exposed to moderate temperature regimes of 20/30°C and 15/25°C reaching 94.83%, and 94.30% respectively. This was followed by 25/35°C (93.30%), 10/20°C and (92.78%) respectively while other regimes showed recorded median germination, except 5/5°C, 5/10°C, 5/40°C, 5/35°C, and at 35/40°C, 40/40°C regimes the germination of *C*. *bonariensis* seeds was stopped completely. The extended T_50_ was estimated by 22.54 days at 5/10°C, and the germination rate (G_rate_) was found to be considerably greater at 20/30°C by 0.457. The seeds of *P*. *hysterophorus* were examined for germination across all temperature regimes. The maximum germination percentage (G_max_) of *P*. *hysterophorus* was found at temperature regimes of 20/25°C (96.28%), and subsequently by 25/30°C (91.94%), and 25/35°C (90.94%), respectively. Nonetheless, the germination did not occur in both low and high-temperature regimes of 5/5°C, 5/10°C, 5/35°C, 5/40°C, 10/35°C, 10/40°C, 35/35°C and 40/40°C. The prolonged half-time of seed germination time (T_50_) was detected at 15/40°C by 28.72 days. While higher germination rate (G_rate_) was significantly noticed at 10/20°C by 0.847 along all tested regimes.

**Table 1:** Germination pattern of *B*. *pilosa*, *C*. *bonariensis* and *P*. *hysterophorus* under alternating temperature regimes and 12 h/12h (dark and light).

	*B*. *pilosa*			*C*. *bonariensis*			*P*. *hysterophorus*		
Temperature (°C)	G _(max)_	G _(rate)_	T _(50)_	G _(max)_	G _(rate)_	T _(50)_	G _(max)_	G _(rate)_	T _(50)_
5 / 5	0.00^n^	0.00	0.00	0.00 ^g^	0.00	0.00	0.000^h^	0.000	0.000
5 / 10	0.00^n^	0.00	0.00	12.34(1.89)^f^	4.406(0.21)	22.54(1.18)	19.20(1.65)^g^	2.833(0.21)	16.78(0.31)
5 / 15	6.27(1.54)^hm^	3.818(0.13)	23.27(0.51)	24.93(1.88)^ef^	2.760(0.15)	19.94(0.48)	29.79(0.62)^g^	2.216(0.05)	25.59(0.07)
5 / 20	22.48(1.41)^km^	15.98(0.31)	2.798(0.028)	49.90(1.89)^de^	3.824(0.21)	13. 87(1.18)	48.40(0.63)^f^	2.144(0.20)	18. 49(0.22)
5 / 25	30.09(1.81)^fh^	2.32(0.15)	12.869(0.14)	43.26(2.29)^de^	3.916(0.22)	7. 97(0.72)	68.40(0.72)^de^	1.248(0.16)	18.32(0.08)
5 / 30	44.45(1.61)^gfh^	2.146(0.15)	10.378(0.21)	21.12 (1.53)^ef^	5.289(0.22)	18.86(1.23)	27.45(0.42)^g^	2.076(0.07)	18. 64(0.07)
5 / 35	10.81(3.47)^hm^	4.191(0.23)	21.82(0.86)	15.60(1.09)^f^	3.448(0.16)	13.781(0.27)	0.000^h^	0.00	0.00
5 / 40	0.00^n^	0.00	0.00	^0.00 g^	0.00	0.00	0.000^h^	0.00	0.00
10 / 10	1.43(0.41)^hm^	3.863(0.17)	22.172(0.29)	19.81(1.87)^ef^	2.917(0.23)	15.82(0.25)	17.20(1.65)^g^	2.733(0.21)	16.78(0.31)
10 / 15	18.44(1.92)^km^	5.644(0.27)	22.98(1.67)	55.84(1.76)^de^	2.968(0.21)	12. 97(0.55)	59.53(0.67)^ef^	3.448(0.14)	17.75(0.11)
10 / 20	33.76(1.87)^fh^	4.112(0.21)	16.33(0.68)	92.78(0.7) ^ab^	0.658(0.11)	4.16(0.21)	76.45(1.63)^cd^	0.847(0.15)	16.143(0.21)
10 / 25	74.24(0.68)^cd^	1.293(0.19)	8.95(0.21)	83.93(0.87)^bc^	1.986(0.20)	6. 71(0.14)	72.53(1.31)^d^	3.37(0.10)	13.74(0.14)
10 / 30	48.68(1.43) ^fgh^	1.613(0.10)	9.99(0.32)	68.62(0.83)^cd^	2.674(0.08)	5.247(0.10)	37.57(1.11)^g^	2.69(0.24)	20.69(0.31)
10 / 35	9.42(0.48)^hm^	2.28(0.05)	11.54(0.07)	21.54(2.53)^ef^	2.735(0.17)	11.66(0.17)	0.000^h^	0.00	0.00
10 / 40	1.99(0.27)^m^	3.137(0.22)	22.58(0.34)	9.93(0.34)^f^	3.355(0.21)	9. 85(0.34)	0.000^h^	0.00	0.00
15 / 15	40.07(0.74)^fgh^	3.978(0.18)	9.02(0.29)	35.83(0.54)^de^	2.879(0.15)	6.20(0.25)	43.23(1.65)^ef^	2.423(0.18)	8. 21(0.22)
15 / 20	66.74(1.14)^def^	2.665(0.11)	10.20(0.25)	84.89(0.74) ^bc^	0.894(0.16)	4.633(0.27)	70.48(1.90)^cd^	2.304(0.28)	9.35(0.32)
15 / 25	86.808(0.98)^bc^	1.575(0.13)	6.78(0.15)	94.30(1.17) ^ab^	1.193(0.22)	4. 81(0.13)	85.84(1.30)^bc^	1.952(0.15)	8.72(0.10)
15 / 30	38.74(0.88) ^gfh^	1.526(0.14)	6.68(0.15)	74.56(0.64) cd	1.729(0.06)	4.96(0.05)	81.56(1.13)^bc^	0.985(0.05)	5.77(0.06)
15 / 35	54.71(0.67)^gh^	1.820(0.12)	11.47(0.26)	82.36(1.38) ^bcd^	1.727(0.25)	6.46(0.17)	24.54(0.61)^g^	2.19(0.10)	21.23(0.14)
15 / 40	11.73(0.37) ^hm^	3.875(0.39)	14.81(0.38)	34.83(2.52)^de^	2.626(0.15)	11.092(0.48)	6.26(1.19)^h^	3.94(0.27)	28.72(1.43)
20 / 20	11.40 (0.97)^hm^	2.934(0.15)	6.76(0.27)	63.113(0.39) ^cd^	2.828(0.13)	4.45(0.23)	5.48(1.34)^h^	3.268(0.23)	6.68(0.24)
20 / 25	84.61(0.78)^bc^	1.243(0.19)	5.56(0.12)	85.99(2.23)^bc^	1.422(0.15)	8.49(0.37)	96.28(1.39)^a^	0.842(0.02)	5.442(0.03)
20 / 30	88.41(0.75)^bc^	0.954(0.06)	4. 71(0.08)	94.83(0.26)^abc^	0.457(0.11)	4. 72(0.22)	80.813(1.43)^bcd^	2.18(0.18)	10.84(0.21)
20 / 35	75.47(0.89)^cd^	2.227(0.15)	8. 42(0.28)	87.87(1.22) ^bc^	0.950(0.03)	4.06(0.12)	86.32(0.99)^bc^	1. 25(0.21)	6.73(0.11)
20 / 40	32.42(0.69)^fh^	1.857(0.04)	11.97(0.18)	63.26(1.31) ^cd^	1.892(0.14)	5.265(0.29)	54.63(1.93)^de^	1.986(0.21)	8.63(0.25)
25 / 25	29.75(0.48) ^fh^	1.849(0.03)	15.21(0.03)	21.87(1.30) ^f^	2.655(0.04)	3.063(0.23)	57.77(0.88)^ef^	1.923(0.06)	5.15(0.07)
25 / 30	92.94(0.91)^a^	0.619(0.03)	4.91(0.01)	80.48(1.41)^bcd^	0.661(0.03)	3. 95(0.14)	91.15(0.54)^ab^	0.874(0.13)	4.42(0.14)
25 / 35	80.32(1.51)^bc^	0.874(0.15)	4.70(0.16)	83.30(0.82)^bcd^	0.792(0.03	4.74(0.13)	90.94(0.97)^ab^	0.950(0.17)	4.55(0.08)
25 / 40	78.93(0.87)^bc^	1.369(0.15)	3.33 (0.17)	69.36(1.25) ^cd^	0.593(0.02)	4.12(0.22)	60.71(0.93)^de^	1.788(0.05)	4.48(0.07)
30 / 30	9.22(0.74) ^hm^	2.955(0.04)	14.56(0.28)	20.07(1.09)^ef^	2.677(0.02)	14.12(0.02)	27.928(2.30)^g^	6.793(0.23)	15.63(0.76)
30 / 35	91.94(0.76)^a^	1.693(0.10)	5.76(0.12)	59.45(0.57)^de^	1.988(0.08)	5.38(0.09)	50.97(1.72)^f^	2.441(0.16)	5.61(0.69)
30 / 40	85. 90(1.25)^bc^	1.103(0.11)	4.53(0.15)	45.26(0.59)^de^	2.380(0.14)	5.36(0.15)	48.41(3.38)^gf^	2.775(0.26)	9.98(0.90)
35 / 35	0.00^n^	0.00^n^	0.00^n^	1.97(1.15)^f^	4.463(0.06)	8.88(0.17)	0.000^h^	0.00	0.00
35 / 40	8. 20(0.86) ^hm^	3.343(0.14)	7.80(0.23)	0.00^g^	0.00	0.00	0.000^h^	0.00	0.00
40 / 40	0.00^n^	0.00	0.00	0.00^g^	0.00	0.00	0.000^h^	0.00	0.00
F (p values)	218.927(0.000)			142.074(0.00)			76.632(0.00)		
	G _(max) = maximum germination_			G _(rate) = germination rate_			T _(50) = 50% of seed germinated seeds_		

Table Values in parenthesis indicate standard deviations.

In five consequences grouped of temperatures Table 2, the low-temperature regime was more appropriate for *P*. *hysterophorus* than *C*. *bonariensis* to germinate well and not appropriate for *B*. *pilosa*. While the germination of *P*. *hysterophorus* and *C*. *bonariensis* species was limited in the large high and low-temperature regime while this regime is appropriate for *B*. *pilosa* to germinate. In a moderate temperature regime, the germination of *C*. *bonariensis* and *P*. *hysterophorus* was favored to achieve the maximum germination than *B*. *pilosa*. In the high-temperature regime, the highest germination was exhibited from *B*. *pilosa*. Compared to other species, *B*. *pilosa* showed exceptional germination in an extremely high-temperature regime. The maximum germination of the species was detected in a moderate temperature regime for *P*. *hysterophorus* at 20/25°C and *C*. *bonariensis* at 20/30°C respectively, while, *B*. *pilosa* optimum germination was in high temperature regime at 25/30°C. These classifications of *P*. *hysterophorus*, *B*. *pilosa*, and *C*. *bonariensis* seed germination were characterized by their ability over different temperature ranges.

**Table 2:** Average of the three invasive weeds germination percentages over tested temperature ranges.

Temperature ranges	*B*. *pilosa*	*C*. *bonariensis*	*P*. *hysterophorus*
Low-temperature	0.47 ± 0.57	10.71 ± 2.86	12.13 ± 4.44
Large range high and low-temperature	48.02 ± 4.11	18.64 ± 3.18	5.61 ± 1.72
Moderate high and low-temperature	47.86 ± 6.86	68.63 ± 5.74	62.00 ± 2.48
High-temperature	58.56 ± 4.65	55.12 ± 3.77	53.82 ± 2.48
Extremely high-temperature	2.73 ± 1.29	0.63 ± 0.93	0.00 ± 0.00
Optimum germination regime	25/30°C	20/30°C	20/25°C
F (p value)	17.654 (0.00)	29.65 (0.00)	11.551 (0.00)

### Germination abilities of invasive *B*. *pilosa*, *C*. *bonariensis*, and *P*. *hysterophorus* under environmental stress

The osmotic pressure (OP) was investigated in the range of -0.1 to -0.5 MPa in invasive *B*. *pilosa*, *C*. *bonariensis* and *P*. *hysterophorus*, seeds germination under laboratory conditions Table 3. As for *B*. *pilosa*, the osmotic pressure established a significant (F = 71.21, P ≤ 0.00) impact on germination, while these reduction percentages ranged from 17.1 to 95.7%, over the control, whereas, it cannot germinate in -1.5 MPa concentration of OP. Furthermore, Furthermore, the G_50_ of the germinated seeds was achieved by the tested potentials of osmotic reaching -0.406 MPa (R^2^ = 0.986). Accordingly, *B*. *pilosa* is more osmotic pressure tolerant than other species. Finally, the osmotic pressure had a substantial impact on the germination of *C*. *bonariensis* seeds (F = 98.8, P < 0.00), with no germination occurring at -1.5 MPa. These osmotic pressure treatments showed a percentage decrease over the control range from 5.9 to 91.2% in seed germination. Furthermore, 50% of the germinated seeds (G_50_) responded to the osmotic potentials recognized at -0.579 MPa. (R^2^ = 0.996). Regarding *P*. *hysterophorus*, osmotic pressure was shown to have a significant influence (F = 25.39, P ≤ 0.00) and decreased germination percentage ranging from 22.7 to 97% as compared to the control. Moreover, germination has not taken place at -1 or -1.5 MPa. The half germination percentage (G_50_) affected by osmotic potentials was -0.32 MPa (R^2^ = 0.989). As a result, it has enough resistance to water deficiency but *P*. *hysterophorus* cannot germinate under droughty circumstances. Also, *C*. *bonariensis* is more drought-tolerant compared with *P*. *hysterophorus*.

**Table 3:** Effect of Osmotic pressure in *B*. *pilosa*, *C*. *bonariensis* and *P*. *hysterophorus* seeds germination percentage.

Osmotic* *potentials	*B*. *pilosa*		*C*. *bonariensis*		*P*. *hysterophorus*	
MPa	Germ. %	R %	Germ. %	R %	Germ. %	R %
0.00	87.50^a^ ±1.16	0.00	85.00 ^a^ ± 2.82	0.00	82.50^a^ ± 1.29	0.00
-0.10	65.00^b^ ± 2.82	28.77	80.00^b^ ± 1.29	14.71	63.75^b^ ± 0.85	36.00
-0.25	64.25^b^ ± 2.96	54.79	68.25 ^c^ ± 0.82	35.29	60.00^b^ ± 0.96	82.00
-0.50	41.25^c^ ± 1.58	80.82	55.00^d^ ± 0.94	72.06	2.50^c^ ± 0.00	100.00
-1.00	3.75^d^ ± 0.60	93.15	7.50^e^ ± 0.50	95.59	0.00^d^ ± 0.00	100.00
-1.50	0.00	100.00	0.00	100.00	0.00	100.00
F (p value)	71.21(0.00)		98.80(0.00)		25.39(0.00)	

The germination of invasive *P*. *hysterophorus*, *B*. *pilosa*, and *C*. *bonariensis* seeds appeared to be influenced by salinity (sodium chloride) in proportion to the applied concentration Table 4. As for *B*. *pilosa*, seed germination was significantly decreased (F = 275.13, P ≤ 0.00) up to a concentration increased. But seed germination dropped sharply from 3000 to 4000 ppm and stopped entirely at 5000 ppm of NaCl. The measured concentration resulted in a 50% inhibition by 2878.35 ppm of NaCl, (R^2^ = 0.956). Finally, *C*. *bonariensis* seed germination was affected by NaCl concentration significantly (F = 218.9, P ≤ 0.000). While it’s negligible germination at 3000 ppm, and the germination didn’t occur over 4000 ppm. It yielded an estimated G_50_ of 2490.9 ppm, (R^2^ = 0.965). Regarding *P*. *hysterophorus*, seed germination showed a significant fluctuation (F = 145.34, P ≤ 0.00) from increasing the doses to 500 ppm of sodium chloride which completely stopped the germination. There was a slight decrease in seed germination between 1000 and 2000 ppm, but, there was a sharp decline between 3000 and 4000 ppm. Lastly, the concentration caused 50% inhibition (G_50_) was 2490.8 ppm of NaCl, (R^2^ = 0.999).

**Table 4:** Effect of Salinity in *B*. *pilosa*, *C*. *bonariensis* and *P*. *hysterophorus* seeds germination percentage.

NaCl (ppm)	*B*. *pilosa*	*C*. *bonariensis*,	*P*. *hysterophorus*.
0	58.75^c^ ± 0.90	62.50^b^ ± 1.29	65.00^a^ ± 0.82
500	91.25^a^ ±1.86	91.25^a^ ± 0.95	68.75^a^ ± 0.96
1000	91.25^a^ ±1.6	65.00^b^ ±1.82	63.75^b^ ± 0.96
2000	82.50_b_ ± 0.58	53.75^bc^ ±1.90	51.25^bc^ ± 1.26
3000	28.75^c^ ± 1.16	21.25^d^ ± 0.96	15.00^c^ ± 0.82
4000	8.75^d^ ± 0.50	0.00^e^ ± 0.00	1.25^d^ ± 0.50
5000	0.00	0.00	0.00
F (p value)	275.13(0.00)	218.9(0.00)	145.34(0.00)

The study investigated the germination of invasive *P*. *hysterophorus*, *B*. *pilosa*, and *C*. *bonariensis* weeds under varied pH conditions Table 5. As for *B*. *pilosa*, germination was significantly affected (F = 39.90, P ≤ 0.000), with pH 6 showing the highest germination percentage of 87.5%. However, at 1, 2, 3, 11, 12 pH levels, germination of seeds did not occur and their G_50_ was determined to be at pH 4.03, (R^2^ = 0.782). *C*. *bonariensis* germination was affected significantly (F = 32.5, P ≤ 0.00). However, germination did not occur at pH of 1, 2, 3, 11, 12 levels. This weed recorded the optimum germination at pH 7 by 80%, as well as G_50_ by pH 4.18, (R^2^ = 0.832). According to the results, this weed had the best germination at pH 7 by 80% and G_50_ at pH 4.18. The findings showed a significant impact of pH on the germination of *P*. *hysterophorus* seeds (F = 57.64, P < 0.00). The greatest germination percentage of 85% was obtained at pH 7. Unfortunately, germination does not occur for pH values of 1, 2, 3, 11, or 12. Furthermore, the model projected the half germination value (G_50_) to be pH 4.42, (R^2^ = 0.892).

**Table 5:** Effect of pH levels in *B*. *pilosa*, *C*. *bonariensis* and *P*. *hysterophorus* seeds germination percentage.

pH	*B*. *pilosa*	*C*. *bonariensis*	*P*. *hysterophorus*
1	0.000	0.000	0.00
2	0.000	0.000	0.00
3	0.000	0.000	0.00
4	11.25^d^ ± 1.25	11.25^c^ ± 1.25	5.00^d^ ± 0.54
5	75.00^ab^ ± 6.33	15.00^c^ ± 0.74	31.25^c^ ± 2.88
6	87.50^a^ ± 1.46	73.75^a^ ± 3.50	73.75^a^ ± 4.63
7	60.00^b^ ± 3.01	80.00^a^ ± 1.06	85.00^a^ ± 1.19
8	48.75^c^ ± 1.46	27.50^b^ ± 3.48	63.75^ab^ ± 2.13
9	27.50^d^ ± 2.50	5.00^d^ ± 0.60	18.75^c^ ± 2.86
10	2.50^d^ ± 2.83	0.00^d^ ± 0.76	3.75^d^ ± 1.67
11	0.000	0.000	0.000
12	0.000	0.000	0.000
F (P value)	32.50(0.00)	39.90 (0.00)	57.64(0.00)

### Growth rate of invasive *B*. *pilosa*, *C*. *bonariensis*, and *P*. *hysterophorus* during the stages in invaded lands

The RGR of *B*. *pilosa*, *C*. *bonariensis*, and *P*. *hysterophorus* invasive species was significantly high due to the cumulative accumulation of Total mass of dry weight from the initial growth to the seeds stage. The higher RGR indicated their advantage in competition with other plants after germination and then developed sluggishly in the interval of juvenile-flowering. These are followed by the low rate of growth in the intervals of flowering-seeds. Among the investigated invasive species, the RGR was significantly higher in (F = 3220.12, p ≤ 0.000) *B*. *pilosa*, and (F = 13535, p ≤ 0.00) C. *bonariensis* in the summer season than (F = 4898.61, p ≤ 0.00) *P*. *hysterophorus* in winter in invaded fields. On the other side, the NAR through the development stages was significantly increased in *B*. *pilosa* (F = 1759.85, p ≤ 0.000), *C*. *bonariensis* (F = 1411.16 p ≤ 0.000), and *P*. *hysterophorus* (F = 3993.67, p ≤ 0.000) respectivel*y*. This increase is associated with increasing LAR and SLA from the seedling stages to reach the optimum at juvenile *B*. *pilosa*, *C*. *bonariensis* in summer and reach the maximum in *P*. *hysterophorus* in the flowering stage during the winter season Table 6.

**Table 6:** The tested invasive species RGR and NAR during growth stages.

	RGR (mg per day)			NAR (mg mm^−2^ day−1)		
Growth stages	*C*. *bonariensis*	*B*. *pilosa*	*P*.*hysterophorus*	*C*. *bonariensis*	*B*. *pilosa*	*P*. *hysterophorus*
Seedling	170.06 ± 2.64	164.97 ± 3.28	167.22 ± 2.36	25.22±1.57	11.89 ± 1.78	7.18 ±1.66
Juvenile	35.59 ± 3.18	29.84 ± 2.87	33.39 ± 4.42	130.63 ± 5.55	60.90 ± 3.29	9.47 ± 1.98
Flowering	24.21 ± 1.15	42.65 ± 1.14	33.77 ± 1.06	53.70 ±1.69	18.50 ± 0.87	141.22 ± 3.72
Seeds	19.103 ± 1.07	14.851 ± 0.53	16.789 ± 0.70	35.40 ±1.33	12.69 ± 0.61	12.49 ± 0.88
F	3220.12(0.00)	13535.46(0.00)	4898.61(0.00)	1759.85(0.00)	1411.16(0.00)	3993.67(0.00)

± = Standard deviation, RGR = Relative growth rate, NAR = Net Assimilation Rate.

Data in Table 7 showed that morphological traits of *B*. *pilosa*, *C*. *bonariensis*, and *P*. *hysterophorus* were distinguished during their life stages in different soil types and composition. The species showed a noteworthy increase in growth and development when compared to the following parameters: plant height (F = 48.69, p ≤ 0.000), number of leaves (F = 320.76, *p* ≤ 0.000), weight (dry) of the stems (F = 37.94, *p* ≤ 0.000), weight (dry) of leaf (F = 791.182, p ≤ 0.00), and the overall dry weight (F = 57.28, p ≤ 0.00), respectively. The highest plasticity index (PI) was observed in the accumulation of dry matter during the development stages. The plasticity was attained 0.968 (*C*. *bonariensis*), 0.985 (*B*. *pilosa*) and 0.957 (*P*. *hysterophorus*) respectively. Conversely, the lowest plasticity values were found in the specific stem length of *C*. *bonariensis* (0.440), root mass fraction of *B*. *pilosa*, (0.467) and shoot/ root ratio of *P*. *hysterophorus* (0.340), respectively.

**Table 7:** Invasive species Plasticity index during growth stages and invaded soils chemical and physical analysis.

Plant parts during growth stages	Plasticity index (PI)			Soil analysis	El-Behria	Al-Qalyubia	El-Ghrabia
	*C*. *bonariensis*	*B*. *pilosa*	*P*. *hysterophorus*		*C*. *bonariensis*	*B*. *pilosa*	*P*. *hysterophorus*
Total dry mass (mg)	0.968	0.985	0.957	pH	8.100	8.000	7.600
Plant Length (cm)	0.910	0.941	0.911	EC (ds/m)	0.827	1.454	0.235
Leaves area (cm^2^)	0.958	0.948	0.820	TDS (mg/L)	2.477	1.234	0.312
Number of leaves	0.946	0.941	0.956	Ca (meq/L)	12.390	3.342	2.255
Specific stem length	0.440	0.451	0.362	Mg (meq/L)	11.146	2.134	0.452
Shoot/ root ratio	0.462	0.467	0.340	Na (meq/L)	50.721	6.035	2.014
Root mass fraction	0.529	0.448	0.364	K (meq/L)	1.450	1.200	0.480
Specific leaf area	0.795	0.717	0.589	HCO_3_ (meq/L)	2.450	1.535	2.783
				SO_4_ (meq/L)	20.625	2.743	4.456
				Cl (meq/L)	6.805	9.700	1.798
				Soil texture	Sandy loam	Clay soil	Clay soil

This research deals with the germination and growth traits of invasive *B*. *pilosa*, *C*. *bonariensis* and *P*. *hysterophorus* species that examined under various environmental conditions to understand their invasion mechanisms. However, there is limited information belonging to these invasive species ecology and behavior in invaded areas.

Under the tested temperature regimes, G _max_, G_rate,_ and T_50_ (half time) for the species *B*. *pilosa*, *C*. *bonariensis*, and *P*. *hysterophorus* varied significantly. The maximum germination was detected at 20/25°C by 96.28% (*P*. *hysterophorus*), 20/30°C by 94.83% (*C*. *bonariensis*), and at 25/30°C reached 92.94% (*B*. *pilosa*) respectively. The preferred regimes for *C*. *bonariensis* and *P*. *hysterophorus* were moderate temperature and for *B*. *pilosa* was a high-temperature regime for suitable germination. However, *P*. *hysterophorus* species did not germinate in an extremely high-temperature regime. The speed of tested invasive species germination over the invaded regions was compared using the half-time of seed germination time (T_50_) and germination rate (G _rate_) values. The better responses were shown in *P*. *hysterophorus* germination at 20 and 25°C in minor time [65] and achieved the maximum at 25–30°C/15-20°C of alternating temperature regimes [66]. The favorite temperatures to *B*. *pilosa* attained 92 & 93% (25/15°C & 30/20°C) than 79% of germination at 35/25°C, while germination failed at 250 mM of NaCl and diminished from 89% to 2% in response to 0.00 to −0.60 MPa of osmotic potential, respectively [67]. The alternating temperatures of 15/25, 20/30, 18/22, 16/24, 14/26, 12/28 and 10/30°C can positively affect the total germination of *C*. *bonariensis* with high germination percentage [68]. These supported our result that found the greatest adaptability and adjacent tendency of *P*. *hysterophorus* and *C*. *bonariensis* to germinate in moderate temperature, while *B*. *pilosa* species can germinate on a wide scale of temperature ranges including extreme temperature regimes. Therefore, *B*. *pilosa* has a good chance to invade new areas and distribute in new habitats. While, the spreading of *C*. *bonariensis* may be in the northern and middle regions of Egypt due to the low temperature condition. Similarly, *P*. *hysterophorus* tended to spread and invade in the northern regions. These results did not completely match with [69,70] they found the response of Asteraceae species germination was greater at 30/20°C similarly to 35/25°C more than the 25/15°C system. Therefore, understanding *C*. *bonariensis* germination ecology and persistence will schedule the control measures and the management in invaded croplands [71]. While its optimum germination was detected at 22–25°C as reported by **Gnanavel** [72]. In general, the T_50_ decreased with increasing the germination rate and vice versa. Asteraceae can characterized by a wide range of temperatures and a good adaptation [73]. These studies supported our finding that the tested Asteraceae species can germinate in cold and extremely warm temperature regimes, which clarified their tendency to spread outside their isothermal geographical zones. Additionally, the higher germination percentage may contribute to high invasion potential based on the preferred regimes to overcome the geographic barriers, following and responding to global warming in the future to exploit new regions. Germination percentage and timing are very important issues in biological invasions [74,75].

The tested environmental stresses were distinguished in the germination of *B pilosa*, *C*. *bonariensis* and *P*. *hysterophorus*, species based on treatment type of osmotic pressure, salt stress and pH and concentration. Generally, *P*. *hysterophorus* seed germination is affected greatly by osmotic pressure, salt stress, and pH than other species. While *C*. *bonariensis* was more germination tolerant to osmotic potentials (drought), which were completely suppressed at -1.5MPa. Likewise, the response of *B*. *pilosa* germination to the effects of salt stress up to 2000 ppm can described as more tolerant, and vice versa at higher concentrations. Moreover, the germination of the tested species mostly happened within a pH series of 3 to 10, and was gradually preferred around pH values of 6 (*B*. *pilosa*) and 7 (*P*. *hysterophorus)* and 7 (*C*. *bonariensis*) respectively. It appeared that the high level of salinity and temperature degree, and pH (acidic or alkaline) conditions completely suppressed the germination of these species. The abiotic prevailingly conditions control invasions of weed species [76]. *C*. *bonariensis* is moderately tolerant to drought and salinity stress [77]. While *B*. *pilosa* at high salt levels can germinate and prefers a moist environment [55]. *P*. *hysterophorus* ability to grow in different environments is strong [78], and habitats, including disturbed and degraded lands [79] and can tolerate salt [80], and showed remarkable survival during unfavorable periods of progressive water stress [81].

The growth investigation revealed a faster growth of *B*. *pilosa*, and *C*. *bonariensis*, than P. *hysterophorus* invasive species based on higher RGR and NAR which reflect a better competitive and colonizing ability in the invaded area. Therefore, *B*. *pilosa* and *C*. *bonariensis* may have a superior capacity for colonization and competition than *P*. *hysterophorus* in the invaded region. The outstanding traits under stress conditions are high growth and rapid germination for IAS [82]. Therefore, the tested characters covering RGR, NAR, LAR, and SLA can used as predictors of invasiveness [83]. The competition ability of IAS depends on their RGR [84]. Plant invasion may be successful because of their strong growth due to exploitative resource acquisition through high specific leaf areas and superior root allocation [85]. Fast RGA, high SLA, and high LAR in plants are significantly associated with rapid invasiveness of *C capture* [86]. Additionally, the findings indicate the plasticity for *B*. *pilosa*, *C*. *bonariensis*, and *P*. *hysterophorus* that are substantially connected with adaptability features and their invasion capacity. The phenotypic plasticity can considered as one of the enabling mechanisms of IAS colonization in varied areas [87] and to promote their invasion success [88–90]. Invasive plant species are maintained by phenology evolution as a potential process during expansion [91]. This besides the germination is the best predictor of IAS invasion across a broad range of geographical areas [92].

## Conclusions

The results presented the capacity of *B*. *pilosa*, *C*. *bonariensis*, and *P*. *hysterophorus* invasive species for germination under a wide range of temperatures. Also, these species have high phenology traits, relative growth rate, net assimilation rate, and plasticity which allow them to acclimatize toward harsh conditions and facilitate the invasive species spreading and colonization. Based on these adaptive features of *B*. *pilosa*, *C*. *bonariensis*, and *P*. *hysterophorus*, the invasion of these species will extend into new areas. Therefore, every attempt should be taken to prevent their invasion via proactive management methods.

## Supporting information

S1 Data (XLSX)
