# Correction: Potentials of invasive *Bidens pilosa, Conyza bonariensis* and *Parthenium hysterophorus* species based on germination patterns and growth traits

**DOI:** 10.1371/journal.pone.0320693

**Published:** 2025-03-20

**Authors:** Rahmah Al-Qthanin, Asmaa M. Radwan, AbdElRaheim M. Donia, Mohamed A. Balah

In the funding statement, the grant number from the funder Deanship of Scientific Research at King Khalid University is listed incorrectly. The correct grant number is: RGP2/439/45.
